# Effect of Saccharomyces cerevisiae on alfalfa nutrient degradation characteristics and rumen microbial populations of steers fed diets with different concentrate-to-forage ratios

**DOI:** 10.1186/2049-1891-5-24

**Published:** 2014-05-01

**Authors:** Gengzhi Ding, Ying Chang, Liping Zhao, Zhenming Zhou, Liping Ren, Qingxiang Meng

**Affiliations:** 1 State Key Laboratory of Animal Nutrition, College of Animal Science and Technology, China Agricultural University, Beijing 100193, China; 2 Lallemand biology Technology (Beijing) Co. Ltd., Beijing, China

**Keywords:** Concentrate-to-forage ratios, In *situ*, Real-time PCR, Rumen microorganism, Steers

## Abstract

Live yeast (*Saccharomyces cerevisiae)* constitutes an effective additive for animal production; its probiotic effect may be related to the concentrate-to-forage ratio (CTFR). The objective of this study was to assess the effects of *S. cerevisiae* (SC) on fiber degradation and rumen microbial populations in steers fed diets with different levels of dietary concentrate. Ten Simmental × Local crossbred steers (450 ± 50 kg BW) were assigned to a control group or an SC group. Both groups were fed the same basal diet but the SC group received SC supplementation (8 × 10^9^ cfu/h/d through the ruminal fistula) following a two-period crossover design. Each period consisted of four phases, each of which lasted 17 d: 10 d for dietary adaptation, 6 d for degradation study, and 1 d for rumen sample collection. From the 1^st^ to the 4^th^ phase, steers were fed in a stepwise fashion with increasing CTFRs, i.e., 30:70, 50:50, 70:30, and 90:10. The kinetics of dry matter and fiber degradation of alfalfa pellets were evaluated; the rumen microbial populations were detected using real-time PCR. The results revealed no significant (*P* > 0.05) interactions between dietary CTFR and SC for most parameters. Dietary CTFR had a significant effect (*P* < 0.01) on degradation characteristics of alfalfa pellets and the copies of rumen microorganism; the increasing concentrate level resulted in linear, quadratic or cubic variation trend for these parameters. SC supplementation significantly (*P* < 0.05) affected dry matter (DM) and neutral detergent fiber (NDF) degradation rates (*c*_DM_, *c*_NDF_) and NDF effective degradability (ED_NDF_). Compared with the control group, there was an increasing trend of rumen fungi and protozoa in SC group (*P* < 0.1); copies of total bacteria in SC group were significantly higher (*P* < 0.05). Additionally, percentage of *Ruminobacter amylophilus* was significantly lower (*P* < 0.05) but percentage of *Selenomonas ruminantium* was significantly higher (*P* < 0.05) in the SC group. In a word, dietary CTFR had a significant effect on degradation characteristics of forage and rumen microbial population. *S. cerevisiae* had positive effects on DM and NDF degradation rate or effective degradability of forage; *S. cerevisiae* increased rumen total bacteria, fungi, protozoa, and lactate-utilizing bacteria but reduced starch-degrading and lactate-producing bacteria.

## Introduction

Compounds isolated from *Saccharomyces cerevisiae* have been used as antibiotic substitutes to improve cattle production efficiency, especially after antibiotics were banned by the European Union [1]. Studies have shown that the *S. cerevisiae* CNCM I-1077 strain (Levucell SC, Lallemand, Toulouse, France) has positive effects on milk production and daily feed intake of dairy cows and goats [2-4]. The primary mechanisms by which live yeasts affect animal performance appears to be related to the effect of yeast on rumen bacterial populations [5], consequently, on nutrient degradation. However, the effect of live yeast on nutrient degradation in the rumen is highly variable. Guedes et al. [6] reported that supplementing cattle with live yeast increased ruminal neutral detergent fiber (NDF) degradation of maize silage, while Mir and Mir [7] reported that live yeast supplementation had no effects on ruminal dry matter (DM) or NDF degradation. Part of these differences may be attributed to different basal diets, especially levels of dietary concentrates [8,9]. According to previous studies [6], probiotic role of live yeast is relating to its effect on stabilizing ruminal pH. However, ruminal pH is function of the level of concentrate. Hence, the effect of live yeast on nutrient degradation characteristics and rumen bacteria population may be related to concentrate to forage ratios.

Although several studies have reported that *S. cerevisiae* has positive effects on animal production performance, nutrient degradation, and rumen bacterial population, the majority of these studies focused on dairy cattle and small ruminants such as sheep and goats [2-4]. Few studies have focused on the effects of live yeast on nutrient degradation and rumen microbial population of beef cattle fed different levels of dietary concentrates. Therefore, the objective of this study was to investigate the effects of supplementation with *S. cerevisiae* (SC) on nutrient degradation and rumen microbial population of beef cattle fed diets with different concentrate-to-forage ratios.

## Materials and methods

### Animals, diets, and experimental design

Ten Simmental × local crossbred steers (450 ± 50 kg body weight) fitted with 10-cm diameter rumen cannulas were used as experimental animals. This study was approved by the Animal Care and Use Committee of the College of Animal Science and Technology of China Agricultural University (ACUC-CAST, #20120806BCRC004). The steers were individually housed in tie-stall barns, and feed, fresh water were available *ad libitum*. Steers were randomly assigned to one of two groups: a control group, which received the basal diet with no SC supplementation, or an SC group, which received the basal diet with SC supplementation, following a 2-period crossover design. Each period consisted of four phases; each phase lasted 17 d: 16 d for dietary adaptation and 1 d for rumen liquor sampling. From the 1^st^ to the 4^th^ phase, steers were fed with increasing dietary concentrate levels in a stepwise fashion. Between periods, steers were fed low level concentrate diet without any treatment during 65 d as a wash-out period. During the wash-out period, ruminal pH and protozoa number were monitored until which recovered to normal level. The stepwise diets (Table 1) were formulated to meet the nutrient requirements (NRC, 2000), with concentrate-to-forage ratios (CTFR) of 30:70 (Phase 1), 50:50 (Phase 2), 70:30 (Phase 3), and 90:10 (Phase 4). Prior to each phase, the diets were mixed and pressed into high density bales using a specialized wrapping machine (DK-850C, Jintudi Co, Baoding, China). During the experiment, active dry SC (I-1077, Levucell SC, Lallemand, Toulouse, France) at 8 × 10^9^ cfu/h/d was added directly into the rumen through the cannulas just prior to the morning feeding.

**Table 1:** Ingredients and chemical composition of basal diets

**Item**	**Dietary concentrate to forage ratios**			
	**30:70**	**50:50**	**70:30**	**90:10**
Ingredient, % DM				
Steam-flaked maize^1^	13.00	34.00	54.00	74.00
Soybean curb residue^2^	15.20	13.90	13.70	13.30
Maize stalks	57.00	38.00	19.00	1.00
Chinese ryegrass	4.00	5.00	6.00	6.00
Alfalfa pellets	9.00	7.00	5.00	3.00
Salt	0.50	0.50	0.50	0.50
Limestone	0.40	0.70	1.10	1.50
Dicalcium phosphate	0.20	0.20	0.00	0.00
Magnesium oxide	0.20	0.20	0.20	0.20
Sodium bicarbonate	0.50	0.50	0.50	0.50
Composition, % DM				
ME, MJ/kg	9.10	10.30	11.70	12.80
CP	12.70	12.70	12.70	12.80
aNDF	54.20	42.70	31.60	20.80
Starch	12.20	26.80	40.90	54.20
Ca	0.64	0.64	0.63	0.67
P	0.32	0.34	0.33	0.35

^1^Flaking density is 360 g/L.

^2^A by-product comes from soybean processed into bean curd which is a kind of food in China.

### In situ rumen incubation

Alfalfa pellets were passed through a 2-mm screen in a Wiley laboratory mill; 5 g DM was transferred to separate number-coded nylon bags (8 cm × 12 cm) of 38 μm pore size. For incubation purposes, each steer had a total of three bags per sample. Rumen incubations were performed according to the method of Ørskov et al. [10] but following the gradual addition/all out schedule [11]. Samples were incubated in the rumen for 168, 96, 48, 24, 12, 6, and 0 h, respectively; for NDF, the samples were incubated for 240 h. All bags were inserted at the same time (0800 h) just prior to the morning feeding and apart from the 12 h bags and 6 h bags, which were inserted at 0700 h and 1300 h. Following incubation, the bags were removed from the rumen and rinsed with cold tap water to remove excess ruminal contents and to remove microbial activity. The bags were washed with cold water without detergent in a washing machine and dried at 60°C for 48 h. The 0 h incubation samples were only washed. The bags were weighed and residues were pooled according to group and incubation times and passed through a 1-mm screen. DM and NDF were analyzed. Data for DM and NDF disappearance at different incubation times were fitted to the following models [10],

$$p=a+b\times \left(1-e^{–ct}\right)$$

$$\text{ED}=a+b\times c/\left(c+k\right)$$

where *p* is the fraction disappearance at time *t*; *a* is the soluble or rapidly degradable fraction; *b* is the insoluble but potentially degradable fraction; *c* is the rate constant of degradation of potentially degradable insoluble fraction (/h); *t* is the time of rumen incubation (h); *k* is the rumen passage rate (/h), and ED is the effective degradability. In this experiment, *k* = 0.03/h was used [12]. DM in feedstuff and residues was measured by drying the samples at 60°C for 48 h in a forced-air oven. NDF [13] was measured using an ANKOM^200^ Fiber Analyzer (ANKOM Technology Corp., Fairport, NY, USA). NDF was assayed with heat stable amylase (aNDF) and without sodium sulfite; NDF results included residual ash [14].

### Ruminal fluid collection

On the last day of each phase, rumen samples were collected by hand from four locations in the rumen and reticulum through rumen cannulas at 3 h after the morning feeding. Aliquots were filtered through two layers of cheese cloth; 40 mL of the filtered rumen fluid was transferred to 50-mL centrifuge tubes, which were flash frozen in liquid nitrogen, transferred to the laboratory, and stored at −80°C prior to DNA extraction; these steps lasted 10 min.

### DNA extraction and real-time PCR

Total genomic DNA from 200 μL of frozen rumen samples was extracted using TIANGEN® TIANamp Stool DNA Kit (Tiangen Biotech Co., Ltd., Beijing, China). DNA concentration and purity (OD_260/280_ and OD_260/230_, respectively) were determined using the NanoDrop ND-1000 spectrophotometer (NanoDrop Technologies).

Quantitative real-time PCR was performed with the 7300 Real-Time PCR System (Applied Biosystems) using SYBR green chemistry (SuperReal PreMix Plus, Tiangen Biotech Co., Ltd.). The primers of target microorganism are shown in Table 2. DNA extract (1 μL) was added to the amplification reaction (20 μL), containing 0.3 μL of each primer, 7.9 μL of 2× SuperReal PreMix Plus (with SYBR Green), 8 μL of ddH_2_O, and 2.5 μL of 50 × ROX Reference Dye. The thermal cycling conditions consisted of an initial Taq activation step at 95°C for 15 min, followed by 40 cycles of 95°C for 15 s and of 60°C for 30 s, followed by an amplicon dissociation stage (95°C for 15 s and 60°C for 1 min, increasing 0.5°C/cycle until 95°C was reached), which confirmed specificity via dissociation curve analysis of PCR end products. Fluorescence detection was performed at the end of each denaturation and extension step. For robustness, 3 replicates of each DNA sample were analyzed in the same plate.

**Table 2:** Primers used in real-time PCR for the detection of rumen microorganism

**Target bacterium**	**Primer sequence (5'-3')**	**References**
Total bacteria	F, CGGCAACGAGCGCAACCC	[15]
	R, CCATTGTAGCACGTGTGTAGCC	
Rumen fungi	F, GAGGAAGTAAAAGTCGTAACAAGGTTTC	[16]
	R, CAAATTCACAAAGGGTAGGATGATT	
Protozoa	F, GCTTTCGWTGGTAGTGTATT	[16]
	R, CTTGCCCTCYAATCGTWCT	
*Ruminobacter ablus*	F, TGTTAACAGAGGGAAGCAAAGCA	[17]
	R, TGCAGCCTACAATCCGAACTAA	
*Ruminococcus flavefaciens*	F, TGGCGGACGGGTGAGTAA	[17]
	R, TTACCATCCGTTTCCAGAAGCT	
*Butyrivibrio fibrisolvens*	F, ACCGCATAAGCGCACGGA	[17]
	R, CGGGTCCATCTTGTACCGATAAAT	
*Fibrobacter succinogenes*	F, GCGGGTAGCAAACAGGATTAGA	[17], [18]
	R, CCCCCGGACACCCAGTAT	
*Selenomonas ruminantium*	F, CAATAAGCATTCCGCCTGGG	[17], [18]
	R, TTCACTCAATGTCAAGCCCTGG	
*Streptococcus bovis*	F, CTAATACCGCATAACAGCAT	[19]
	R, AGAAACTTCCTATCTCTAGG	
*Ruminobacter amylophilus*	F, CAACCAGTCGCATTCAGA	[19]
	R, CACTACTCATGGCAACAT	
*Lactobacillus*	F, AGCAGTAGGGAATCTTCCA	[20]
	R, CGCCACTGGTGTTCYTCCATATA	

For the absolute quantification of total bacteria 16S rDNA, rumen fungi and protozoa 18S rDNA gene copies, total bacteria, fungi and protozoa rDNA extracts from mixed rumen samples were loaded on a 1% agarose gel and visualized by ethidium bromide staining respectively. The resulting products were purified using the TIANGEN® TIANgel Midi Purification Kit (Tiangen Biotech Co., Ltd.). The purified PCR product was used as a standard whose concentration was measured in the ND-1000 spectrophotometer and converted to concentration using the following equation, DNA (number of molecules) = (NL × A × 10^−9^)/ (660 × n), where NL is the Avogadro constant (6.02 × 10^23^ molecules per mol); A is the molecular weight of the molecule in the standard; and n is the length of the amplicon in base pairs [21]. Standard curves were constructed with purified total bacteria, rumen fungi, and protozoa rDNA when the copies of total bacteria, rumen fungi, and protozoa were measured.

For quantification of individual species, Relative Quantification ΔCT method [22] was used and the total bacteria used as reference. Amplification efficiencies were calculated using serial dilutions and only efficiencies between 90%-110% were taken into consideration.

### Statistical analyses

SAS (1990) software was used for statistical analyses. Nutrient degradation data and rumen microbial population data were analyzed using the MIXED procedure with the following model,

$$\begin{array}{ll} \;Y_{\text{ijkl}}= & \mu +\text{Perio}d_{i}+\text{Treatmen}t_{j}+\text{Die}t_{k}\\ & +\left(\text{Treatmen}t_{j}\times \text{Die}t_{k}\right)+\text{Stee}r_{l}+\epsilon_{\text{ijkl}} \end{array}$$

where *μ* represented the overall mean, *Period* represented the period (1 or 2), *Treatment* accounted for the fixed effect of yeast supplementation, *Diet* represented the fixed effect of different dietary CTFR, *Steer* accounted for the random effect of each individual animal, *ϵ* account for the unexplained random error. Linear, quadratic and cubic responses for dietary CTFR were assessed using orthogonal polynomial contrast statements.

## Results

The results revealed that there were no significant difference between the 2 periods and there were no interactions between dietary CTFR and SC (*P* > 0.1; Tables 3, 4 and 5) for most parameters except *a*_DM_, *c*_NDF_, and copies of *R. ablus*. Therefore, the main effects of diet and SC are discussed independently.

**Table 3:** Effect of SC supplementation on dry matter (DM) degradation characteristics of alfalfa pellets in steers fed diets with different concentrate-to-forage ratios

**Item**	**CTFR**				**SEM**	**SC**		**SEM**	***P*-value**			**Probability**^**3**^		
	**30:70**	**50:50**	**70:30**	**90:10**		**N**^**1**^	**Y**^**2**^		**CTFR**	**SC**	**CTFR × SC**	**Linear**	**Quadratic**	**Cubic**
*a*, %	26.07	29.46	27.08	26.58	0.64	27.46	27.14	0.45	<0.01	0.61	0.09	0.80	0.01	0.03
*b*, %	38.48	35.95	38.99	39.86	0.75	38.18	38.46	0.53	<0.01	0.71	0.43	0.05	0.04	0.04
*c*, %/h	3.50	3.30	3.00	2.47	0.14	2.86	3.29	0.10	<0.01	0.04	0.14	<0.01	0.25	0.96
ED, %	46.54	48.48	46.62	43.76	0.32	45.65	47.06	0.22	<0.01	<0.01	0.14	<0.01	<0.01	0.12

^1^N: without yeast supplementation;

^2^Y: with yeast supplementation;

^3^Linear indicates linear effect of dietary CTFR; quadratic indicates a quadratic effect of dietary CTFR; cubic indicates a cubic effect of dietary CTFR.

**Table 4:** Effect of SC supplementation on neutral detergent fiber (NDF) degradation characteristics of alfalfa pellets in steers fed diets with different concentrate-to-forage ratios

**Item**	**CTFR**				**SEM**	**SC**		**SEM**	***P*-value**			**Probability**^**3**^		
	**30:70**	**50:50**	**70:30**	**90:10**		**N**^**1**^	**Y**^**2**^		**CTFR**	**SC**	**CTFR × SC**	**Linear**	**Quadratic**	**Cubic**
*a*, %	2.32	6.36	2.70	2.99	0.74	3.22	3.96	0.52	<0.01	0.32	0.13	0.67	0.03	<0.01
*b*, %	46.77	44.76	49.29	49.69	0.92	48.11	47.15	0.65	<0.01	0.30	0.35	<0.01	0.24	0.02
*c*, %/h	3.40	2.77	2.55	1.83	0.11	2.52	2.76	0.08	<0.01	0.03	0.04	<0.01	0.95	0.24
ED, %	26.89	27.35	25.28	22.36	0.44	24.70	26.25	0.31	<0.01	<0.01	0.56	<0.01	<0.01	0.47

^1^N: without yeast supplementation;

^2^Y: with yeast supplementation;

^3^Linear indicates linear effect of dietary CTFR; quadratic indicates a quadratic effect of dietary CTFR; cubic indicates a cubic effect of dietary CTFR.

**Table 5:** Effect of SC supplementation on rumen microbial populations and percentage of target species relative to total bacteria of steers fed diets with different concentrate-to-forage ratios

**Item**	**CTFR**				**SEM**	**SC**		**SEM**	***P*-value**			**Probability**^**3**^		
	**30:70**	**50:50**	**70:30**	**90:10**		**N**^**1**^	**Y**^**2**^		**CTFR**	**SC**	**CTFR × SC**	**Linear**	**Quadratic**	**Cubic**
Total bacteria,	6.95	9.62	13.08	6.21	1.09	7.86	10.07	0.77	<0.01	0.05	0.61	0.82	<0.01	0.04
×10^10^ copies/mL														
Rumen fungi,	6.57	5.10	11.80	3.57	0.91	5.99	7.51	0.64	<0.01	0.10	0.22	0.63	<0.01	<0.01
×10^5^ copies/mL														
Protozoa,	4.03	3.64	17.44	7.44	1.47	6.74	9.54	1.04	<0.01	0.06	0.30	<0.01	<0.01	<0.01
×10^5^ copies/mL														
Percentage relative to total bacteria														
*R. flavefaciens*	0.45	0.20	0.28	0.16	0.03	0.25	0.29	0.02	<0.01	0.25	0.87	<0.01	0.07	<0.01
*R. ablus*	0.02	0.01	0.01	0.00	0.00	0.01	0.01	0.00	<0.01	0.63	0.08	<0.01	0.40	0.02
*FibSuc*^4^	1.03	0.39	0.23	0.11	0.09	0.46	0.43	0.06	<0.01	0.72	0.85	<0.01	0.03	0.41
*ButFib*^5^	0.01	0.01	0.00	0.00	0.00	0.01	0.01	0.00	<0.01	0.37	0.61	<0.01	0.68	0.75
*StrBov*^6^	0.03	0.05	0.01	0.01	0.00	0.03	0.02	0.00	<0.01	0.64	0.83	<0.01	<0.01	<0.01
*RumAmy*^7^	0.13	0.14	0.10	0.07	0.01	0.12	0.10	0.01	<0.01	0.01	0.53	<0.01	0.03	0.19
*SelRum*^8^	3.10	3.24	1.80	1.07	0.21	2.04	2.55	0.15	<0.01	0.02	0.46	<0.01	0.09	0.05
*Lactobacillus*^9^	0.02	0.02	0.04	0.05	0.01	0.04	0.03	0.01	<0.01	0.16	0.73	<0.01	0.51	0.14

^1^N: without yeast supplementation;

^2^Y: with yeast supplementation;

^3^Linear indicates linear effect of dietary CTFR; quadratic indicates a quadratic effect of dietary CTFR; cubic indicates a cubic effect of dietary CTFR;

^4^*FibSuc*: *Fibrobacter succinogenes*;

^5^*ButFib*: *Butyrivibrio fibrisolvens*;

^6^*StrBov*: *Streptococcus bovis*;

^7^*RumAmy*: *Ruminobacter amylophilus*;

^8^*SelRum*: *Selnomonas ruminantium*;

^9^*Lactobacillus*: *Lactobacillus* species*.*

### Effect of diet on alfalfa pellet degradation

The degradation characteristics of alfalfa pellet DM are shown in Table 3. There were statistically significant differences (*P* < 0.01) in *a*_DM_, *b*_DM_, *c*_DM_, and ED_DM_ among the different diets. Increasing concentrate level resulted in a cubic variation trend in *a*_DM_ and *b*_DM_ (*P* < 0.05), and a linear and quadratic decrease in *c*_DM_ and ED_DM_ respectively (*P* < 0.01).

The degradation characteristics of alfalfa pellet NDF are shown in Table 4. Dietary CTFR significantly (*P* < 0.01) affected NDF degradation characteristics and the variation trends were similar to those obtained for DM.

### Effect of SC on alfalfa pellet degradation

There were no significant differences (*P* > 0.05) in *a*_DM_, *b*_DM_, or ED_DM_ of alfalfa pellet between the SC and control groups (Table 3). However, SC supplementation increased (*P* < 0.01) *c*_DM_ of alfalfa pellet compared to the control group.

There were no differences in *a*_NDF_, *b*_NDF_, (*P* > 0.05) between the SC and control groups (Table 4); however, *c*_NDF_ and ED_NDF_ of SC were significantly different from that of the control group (*P* < 0.05).

The relative DM effective degradability had an increasing trend with increasing dietary CTFR and it reached 5.93% (calculated from 45.03%–42.51%)/42.51% × 100%) when dietary CTFR was 90:10.

In addition, the relative NDF effective degradability presented an increasing trend with increasing dietary CTFR and it reached 9.49% (calculated from 23.38%–21. 35%)/21.35% × 100%) when dietary CTFR was 90:10.

### Effect of diet on rumen microbial population

There were significant differences (*P* < 0.01) in rumen microbial population among the four phases (Table 5). With increasing dietary concentrate levels, percentage of *Butyrivibrio fibrisolvens* and *Lactobacillus* species linearly decreased and increased respectively (*P* < 0.01); percentage of *Fibrobacter succinogenes* and *Ruminobacter amylophilus* quadratically decreased respectively (*P* < 0.01); total bacteria, fungi, protozoa and other target bacteria presented cubic variation trend (*P* < 0.05).

### Effect of SC on rumen microbial population

The total bacteria copies in the SC group were significantly higher (*P* < 0.05) than in the control group (Table 5). The copies of rumen fungi and protozoa with SC supplementation increased (*P* < 0.1) compared to the control group. When SC was supplemented, the percentage of *R. amylophilus* and *S. ruminantium* significantly (*P* < 0.05) decreased and increased, respectively. However, there were no significant differences (*P* > 0.05) in other bacterial species.

## Discussion

**Figure 1:**
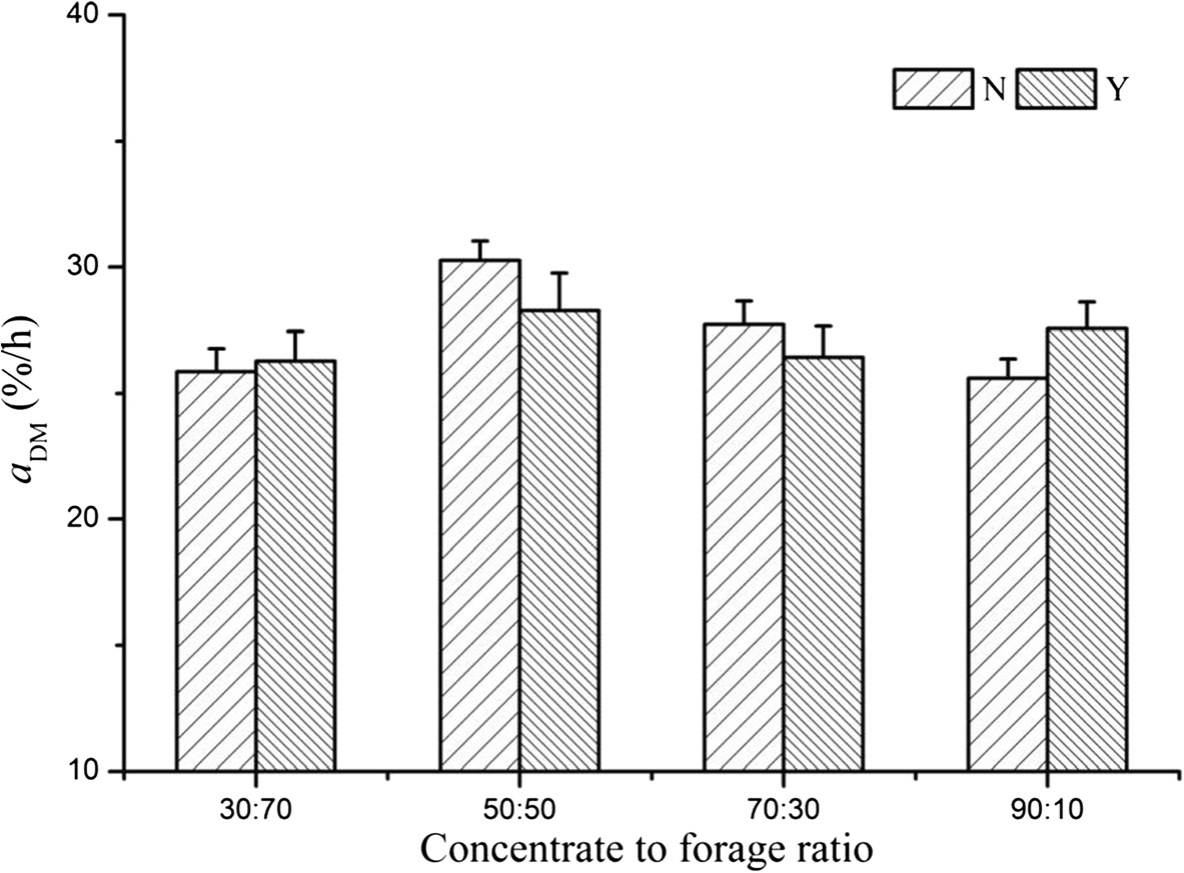
**Effect of SC on *a***_**DM **_**of alfalfa pellet of steers fed different concentrate to forage ratios N: without yeast supplementation; Y: with yeast supplementation.**

**Figure 2:**
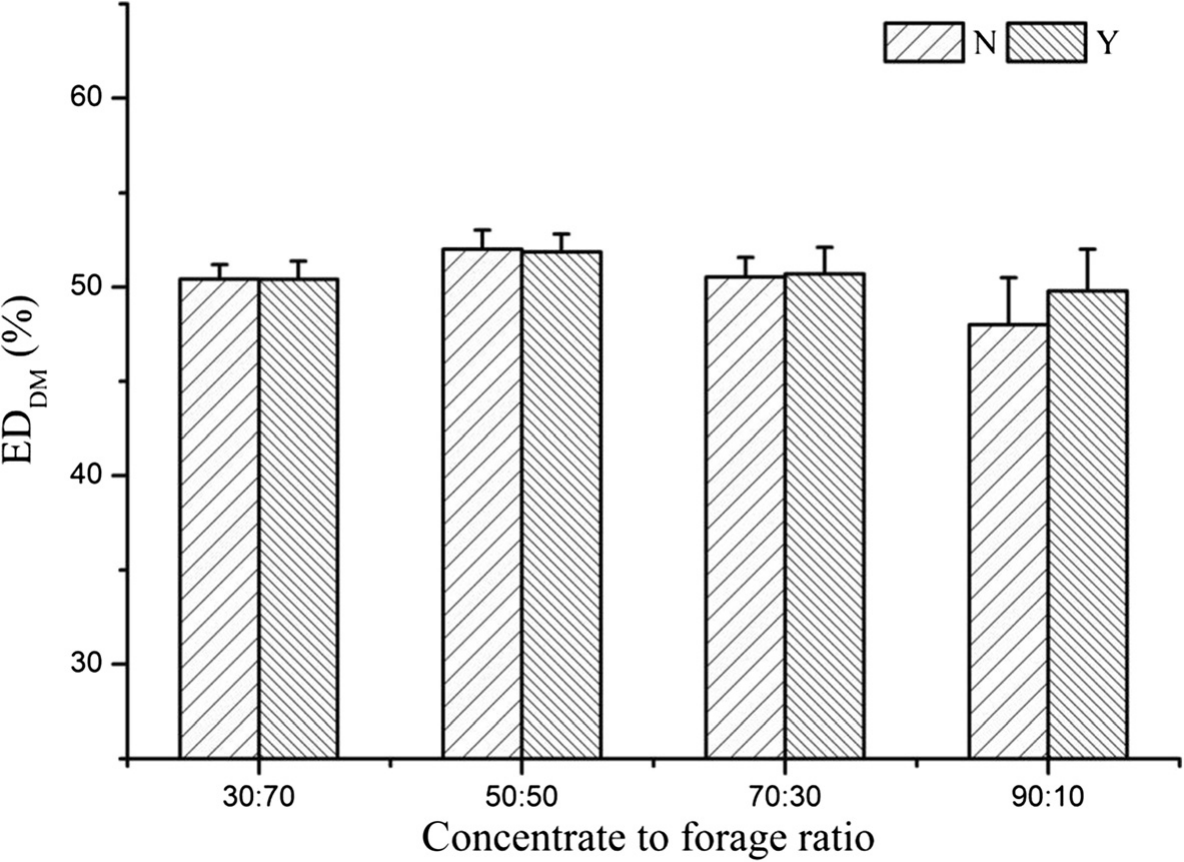
**Effect of SC on ED**_**DM **_**of alfalfa pellet of steers fed different concentrate to forage ratios N: without yeast supplementation; Y: with yeast supplementation.**

**Figure 3:**
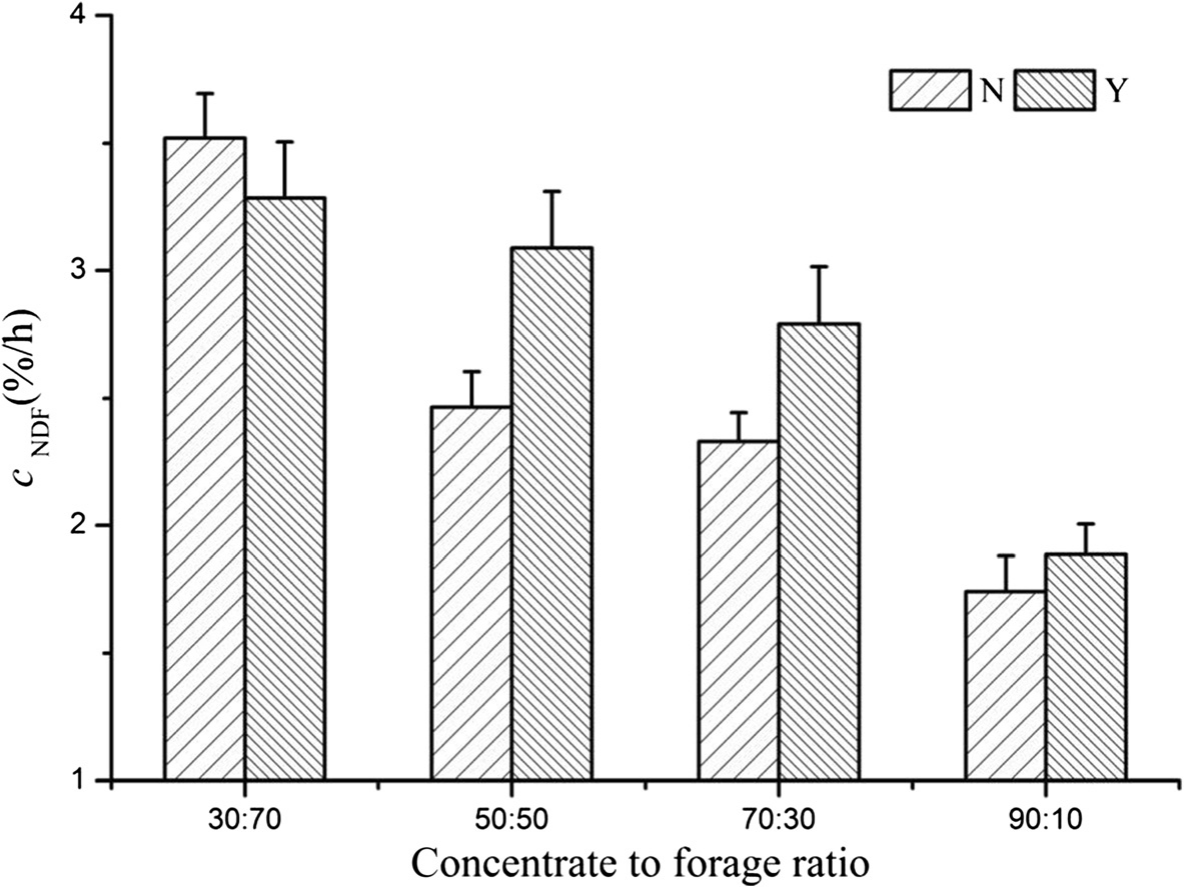
**Effect of SC on *c***_**NDF **_**of alfalfa pellet of steers fed different concentrate to forage ratios N: without yeast supplementation; Y: with yeast supplementation.**

**Figure 4:**
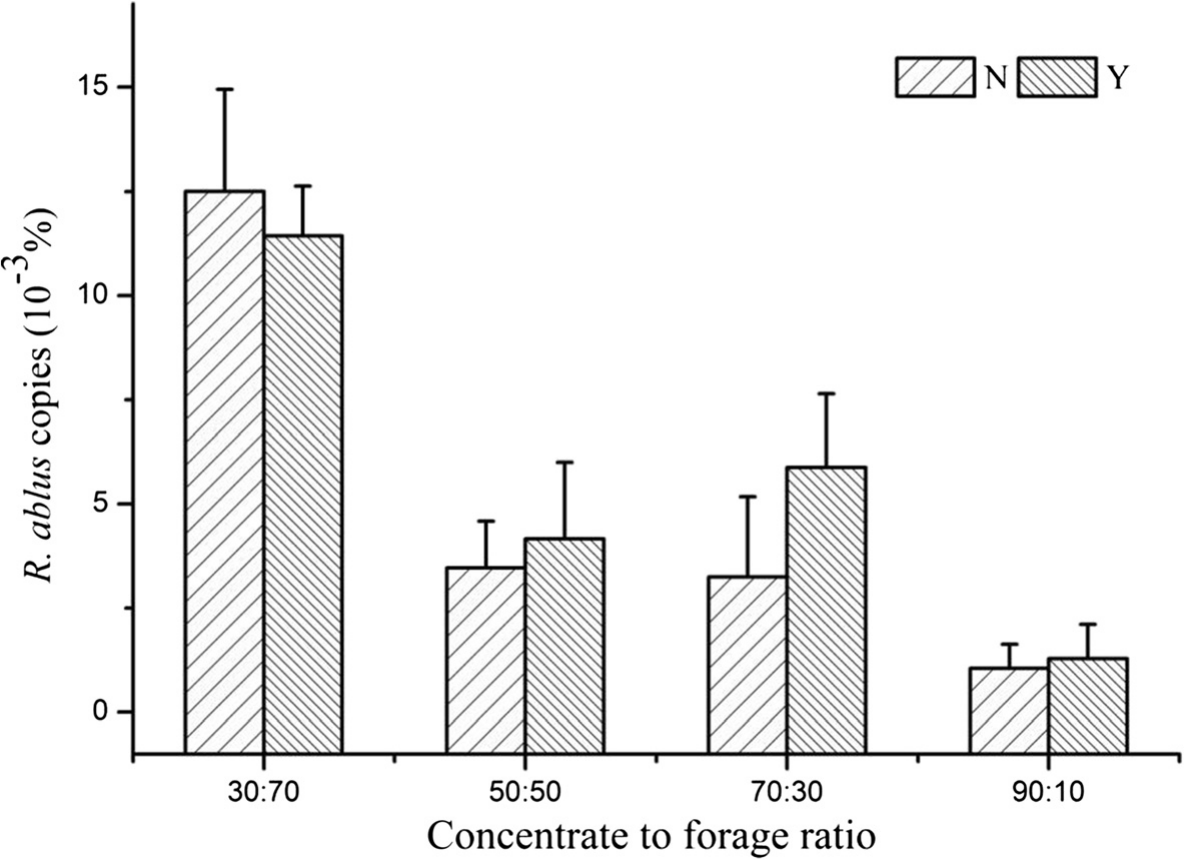
**Effect of SC on percentage of *R. ablus *** **of steers fed different concentrate to forage ratios N: without yeast supplementation; Y: with yeast supplementation.**

There was an interaction of dietary CTFR and SC supplementation on *a*_DM_, *c*_NDF_, and copies of *R. ablus*, which can be illustrated by polynomial contrast results and visually shown in Figures 1, 2, 3 and 4. Without yeast, *a*_DM_ showed quadratic variation trend (Quadratic, *P* < 0.01), with yeast *a*_DM_ presented cubic variation trend (Cubic, *P* < 0.05). Similarly, SC supplementation changed the variation trend of *c*_NDF_, and copies of *R. ablus*. The interaction suggested that SC possess biological effect on fiber degradation, which was related to dietary concentrate to forage ratio.

Roughage is degraded by fiber-degrading bacteria; however, the major fiber-degrading bacteria such as *F. succinogenes*, *R. ablus*, and *R. flavfaciens* are sensitive to low pH values. With increasing dietary concentrate levels, more sugars, starch, and other non-structural carbohydrates are consumed, which contribute to a higher concentration of volatile fatty acids (VFAs). Higher VFA concentrations reduce ruminal pH values and, consequently, the number and activities of fiber-degrading bacteria decreased. Therefore, the effect of dietary CTFR on *a*_DM_/*a*_NDF_, *b*_DM_/*b*_NDF_, *c*_DM_/*c*_NDF_, and ED_DM_/ED_NDF_ can easily be understood. On the other hand, with increasing dietary concentrate levels, the percentage of lactate-producing bacteria such as *S. bovis* and *Lactobacillus* increased; however, lactate-utilizing species such as *S. ruminantium* decreased (Table 5), therefore, lactate may accumulate in the rumen. Consequently, rumen pH declined and total bacteria, fungi, and protozoa levels rapidly declined (Table 5).

When SC was supplemented, total bacteria, fungi, protozoa, fiber-degrading, and lactate-utilizing bacteria increased while starch-degrading and lactate-producing bacteria decreased. Meanwhile, degradation rate or effective degradability of alfalfa DM and NDF improved (Tables 3, 4 and 5). According to Chaucheyras-Durand et al. [5], the probiotic role of SC is attributed to several factors. Firstly, SC possesses the capacity to scavenge unnecessary oxygen in the rumen, reducing the redox potential [23,24]. Therefore, SC contributes to an ecological condition that favors the growth and activities of anaerobic microorganisms and of fiber-degrading bacteria, which improve the DM and NDF degradation rate or effective degradability [6]. Secondly, SC provides thiamin, which is required by fungi for zoosporogenesis [25]. Rumen fungi play important roles in fiber degradation. Thirdly, SC outcompetes starch-degrading bacteria such as *S. bovis* and *Ruminobacter amylophilus* for carbohydrate utilization [5]. As a result, rumen VFA concentration was lower in SC than in the control group [26]. Moreover, SC provides growth factors such as amino acids, peptides, and organic acids, which are essential for lactate-utilizing bacteria [27,28]; consequently, the number of lactate-utilizing bacteria such as *S. ruminantium* improved significantly (Table 5) and the concentration of lactate was reduced [29]. In addition, SC can stimulate rumen protozoa especially ciliate Entodiniomorphid protozoa, which rapidly engulf starch granules and stabilize ruminal pH [3]; the same effect of SC on protozoa was obtained in this study (Table 5). The conclusions of Chaucheyras-Durand et al. (2008) were obtained by in *vitro* experiments, and some experts doubted whether the SC can play the same important role in *vivo* as in *vitro*. This experiment proved that in *vivo* SC really has a positive effect on nutrient degradation and rumen microorganism balance [5].

One the other hand, the effect of SC on rumen nutrient degradation characteristics was inconsistent. For example, results from this and previous studies revealed that SC supplementation improved the degradation rate or effective degradability of forage feedstuff [6,30]. However, other studies revealed that SC supplementation had no effects on DM or NDF degradation [31]. The inconsistent results of SC on forage nutrient degradation characteristics may be related to the dietary concentrate-to-forage ratios.

## Conclusions

Dietary CTFR had a significant effect on degradation characteristics of forage and rumen microbial population. *S. cerevisiae* had positive effects on DM and NDF degradation rate or effective degradability of forage; *S. cerevisiae* increased rumen total bacteria, fungi, protozoa, and lactate-utilizing bacteria but reduced starch-degrading and lactate-producing bacteria.

## Competing interests

The authors declare that they have no competing interests.

## Authors’ contributions

QXM and YC conceived the study and designed the experiment. GZD carried out the statistical analysis and drafted the manuscript. GZD, LPZ, ZMZ and LPR performed the animal experiments and rumen microbial population studies. All authors read and approved the final manuscript.
